# Selection of metallic liquid in sub-6 GHz antenna design for 6G networks

**DOI:** 10.1038/s41598-023-47870-7

**Published:** 2023-11-23

**Authors:** Sasmita Dash, Constantinos Psomas, Ioannis Krikidis

**Affiliations:** Department of Electrical and Computer Engineering, University of Cyprus, Nicosia, Cyprus

**Keywords:** Electronic devices, Electronics, photonics and device physics, Electrical and electronic engineering

## Abstract

The rapid evolution of wireless communication systems toward 6G demands efficient antennas with the features of adaptability and versatility. Liquid antennas have gained significant research interest due to their unique features in realizing small, flexible, transparent, and reconfigurable antennas for promising applications in future wireless systems. In this paper, in order to find a suitable metallic liquid for effective antennas, we design and compare the performance of metallic liquid antennas using Mercury, gallium indium alloy (EGaIn), and Graphene metallic liquid in the sub-6 GHz frequency. The antenna is realized by the metallic liquid in a poly methyl methacrylate microfluidic channel over a liquid crystal polymer substrate at 5.6 GHz frequency. The performance of these metallic liquid antennas is analyzed by their electromagnetic and radiation performance. The Graphene-based metallic liquid antenna shows better electromagnetic performance in comparison to Mercury and EGaIn metallic liquid antennas.

## Introduction

Communication technologies are undergoing a revolutionary shift due to the rapid progress of communication applications. Cellular mobile communication systems have gone through five generations of development. 5G is the latest generation of wireless networks. 5G has already been introduced in some regions of the world and officially became commercially available in 2019, employing sub-6 GHz and mm-wave frequency bands^1^. For a point-to-point link, the mm-wave communication systems generally suffer more loss than sub-6 GHz systems^2,3^. Sub-6 GHz is substantially reasonable for carriers to implement because the range below 6 GHz is longer and penetrates objects in a better way.

In today’s wireless communications system, adaptability and diversified functionality have become the most appealing features of any communications device. The antenna has great importance for the communication system because the design of the air interface largely depends on the design of the antenna. The rapid evolution of wireless communication systems towards 6G demands efficient antennas. As conventional antennas are mostly made up of conductive metals on rigid substrates, they offer remarkable performance but lack mechanical flexibility. Furthermore, bending or stretching these antennas beyond certain limits causes irreversible structural deformation and even destruction^4^. The rigid nature of metallic antennas limits their use in applications that require flexibility. On the other hand, the flowing property and having no deformation limits of metallic liquid make them an ideal alternative for flexible antenna applications. Without sacrificing their electrical properties, liquid metals in microfluidic channels develop extremely flexible and mechanically stable antennas^5^. Modern fabrication techniques for antennas, such as 3D printing, injecting, or spraying metallic liquid onto rigid or flexible substrates, are made possible by the fluidic property of metallic liquids. In contrast to conventional techniques like high-frequency switching, liquid antennas can be easily achieved reconfigurability through electrochemically controlled capillary action or micro pumping. Liquid antennas take advantage of the fluid’s mechanical properties. The presence of metallic liquid in fluidic channels enables the formation of the shape of a fluidic channel due to its low viscosity^6^. Liquid antennas on flexible substrates can bend, fold, stretch, and twist. Therefore, they can stand up to all kinds of mechanical deformation. Due to their high degree of reversibility, they are capable of regaining their original form^7^.

Liquid metal antennas were introduced in the 1990s to make flexible and reconfigurable antennas^8,9^. A comprehensive literature review reveals that Mercury, and alloys of gallium and tin, are utilized in the design of metallic liquid antennas^5,10–12^. Mercury is the first used metallic liquid for antenna design. The fluidic and conductive property of Mercury enables the design of reconfigurable metallic liquid antenna. However, Mercury is toxic in nature and expensive. These facts boost the use of other metallic liquid materials gallium-based alloys^5,11,12^. Alloys based on gallium have remarkable electrical and thermal conductivity, non-toxicity and low viscosity^13,14^. In addition to these properties, gallium indium alloy (EGaIn) has mechanical stability and significantly prevents evaporation^14^. These features of EGaIn help in the improvement of antenna performance without having any effect on the conductivity and efficiency of the antenna. The most recent material that has been reported for antenna design is Graphene. Graphene is emerging as a promising candidate in several applications due to its electrical, thermal, and optical properties. It has excellent material properties for electronic applications. Graphene antennas perform much better than conventional copper metal antennas^15–17^. However, the Graphene metallic liquid for antenna design is not yet found in the literature. Finding the best metallic liquid material for metallic antenna design is vital for the antenna community. This information is pertinent from the perspective of the antenna designer.

In this paper, we design and numerically analyze metallic liquid antennas by using Mercury, EGaIn, and Graphene liquid. We compare the electromagnetic performance of these metallic liquid antennas. A metallic liquid antenna with a working frequency of 5.6 GHz is designed as the candidate antenna to verify the suitability of a specific metallic liquid. In particular, the main contributions of the paper are summarized as follows:

- We consider three antennas using metallic liquids of Mercury (Hg), an alloy of gallium and indium (EGaIn), and Graphene. The metallic liquid is injected in a poly methyl methacrylate (PMMA) microfluidic channel and the antenna is placed over a metallic grounded liquid crystal polymer (LCP) substrate.
- We investigate the numerical analysis of three different metallic liquid antennas and compare the radiation performance of these antennas in terms of gain, efficiency, and bandwidth.
- We design metallic liquid based antennas for sub-6 GHz systems by using the electromagnetic (EM) simulator Ansys HFSS.

## Materials for metallic liquid antennas

Due to their inherent flexibility, liquid materials are a good substitute for stiff or solid conductors in flexible electronics^18^. The fluidic properties of metallic liquids enable a wide range of metallic liquid antennas^5,8–12^. The fluid nature of liquid materials provides additional degrees of freedom to achieve better reconfigurability, even when the radiative elements are created on rigid substrates.

The basis for creating effective liquid antennas is liquid materials. The antenna design and performance mainly result from the properties of the liquid. The liquid materials that are readily available for antenna design can generally be divided into three categories: metallic, non-metallic, and partially metallic liquids. This work primarily focuses only on metallic liquids. Electrical conductivity is typically high in metallic liquids, which is suitable for use as radiating elements in antennas.

Due to their solid-like oxide coating on the surface, metallic liquids give elastomeric antennas with mechanical stability and flexibility. The high conductivity of metallic liquid makes it ideal for antenna applications. The use of metallic liquids as radiative elements enables the design of a considerably highly reconfigurable and flexible antenna than solid conductors like copper. The metallic liquid antenna is ideal for antenna applications due to its high flexibility and deformability, as well as its high conductivity.

The first metallic liquid at room temperature is Mercury (Hg). Alternative metallic liquid materials are mostly in the form of alloys of conductive nanoparticles. Gallium-based alloys exhibit low viscosities, high electrical and thermal conductivities, and non-toxicity. These metallic liquids typically have conductivities of the order of $10^6$ S/m, which is high enough to achieve high radiation efficiency when employed as antenna materials. In this work, Graphene, a new class of new and noble metallic liquid, is introduced in addition to Mercury and gallium-based alloys.

**Table 1:** A comparison of material properties of Mercury, EGaIn, and Graphene.

Metallic liquid	Conductivity (S/m)	Melting point ($^\circ$C)	Density $@ 25^{\circ}$C (g/cm$^{3}$)
Mercury (Hg)	$1.0 \times 10^{6}$	− 38.87	13.55
EGaIn (gallium indium alloy)	$3.4 \times 10^{6}$	16	6.25
Graphene	$50 \times 10^{6}$	Does not melt (sublime at 3600 K)	2.26

### Mercury (Hg)

Mercury is found in its liquid phase at room temperature; Mercury does conduct electricity because it has a metallic property and excellent volume expansion properties with the rise in temperature. The electrons of Mercury are free to move to conduct the flow of electric flux on the surface of the matter. A sufficient amount of electric flux penetrates through the Mercury per unit cross-section area. Mercury can carry electricity when it’s in liquid or molten form. The electrical conductivity of liquid Mercury is due to the transfer of electrons that takes place in the liquid or molten state.

Mercury has a melting point of $-38.87\,^\circ$C, a density $@ 25\,^\circ$C of 13.55 g/cm$^{3}$, and an electrical conductivity of $1.0 \times 10^{6}$ S/m^19^. In addition, due to its high stiction and low oxidation properties, it is a suitable material for the development of a liquid antenna^10^. Mercury, on the other hand, is very toxic and has to be handled very carefully. Therefore, it has limited usage in antenna design.

###  Gallium Indium alloy (EGaIn)

Gallium-based alloys exhibit remarkable electrical and thermal conductivities, low viscosity, and non-toxicity. These materials have a melting point that’s either lower than or near room temperature^13^. One of the most commonly used alloys based on gallium is the EGaIn alloy, which is composed of 75% gallium (Ga) and 25% indium (In). EGaIn has an electrical conductivity of $3.4\times 10^{6}$ S/m and a density of 6.25 g/cm$^{3}$ at $25\,^\circ$C. Furthermore, the melting point of EGaIn is close to room temperature, with a melting point of $16\,^\circ$C^13,14^. The resistivity of EGaIn is $~29.4 \times 10^{-6}$   $\Omega \,$ cm^14^. When the liquid alloy is exposed to air, it reacts with oxygen to create a thin oxide layer on the surface, which enhances mechanical stability and surface tension, and significantly inhibits evaporation. These benefits enhance EGaIn performance in metallic liquid antennas without having any effect on the conductivity and efficiency of antennas. The most significant feature of EGaIn metallic liquid is non-toxicity. The unique feature of EGaIn helps in designing reconfigurable and flexible antennas^5,11,12^.

### Graphene liquid

A carbon-based new metallic liquid material is the liquid form of Graphene. Graphene liquid can be employed for the design of metallic liquid antennas. Because of the high mobility of electrons in the hexagonally arranged carbon atoms of Graphene, it has an electrical conductivity of $\sim 50 \times 10^{6}$ S/m^20^. It is directly dependent on the electron mobility in the matter. The resistivity of Graphene remains between $1.19 \times 10^{-6}$ ohm cm and $1.87 \times 10^{-6}$ ohm cm. It was found that the electrical resistivity of Graphene is an order of magnitude lower than that of the indium-gallium alloy. Graphene liquid is conductive enough to be used as antenna material with high radiation efficiency. Graphene has no known melting point. The reason for this aspect is that it does not melt upon heating. Instead, it undergoes sublimation, which is the transformation of a solid into a gas without ever turning into a liquid. Sublimation of Graphene occurs at temperatures of approximately 3600 K.

**Figure 1:**
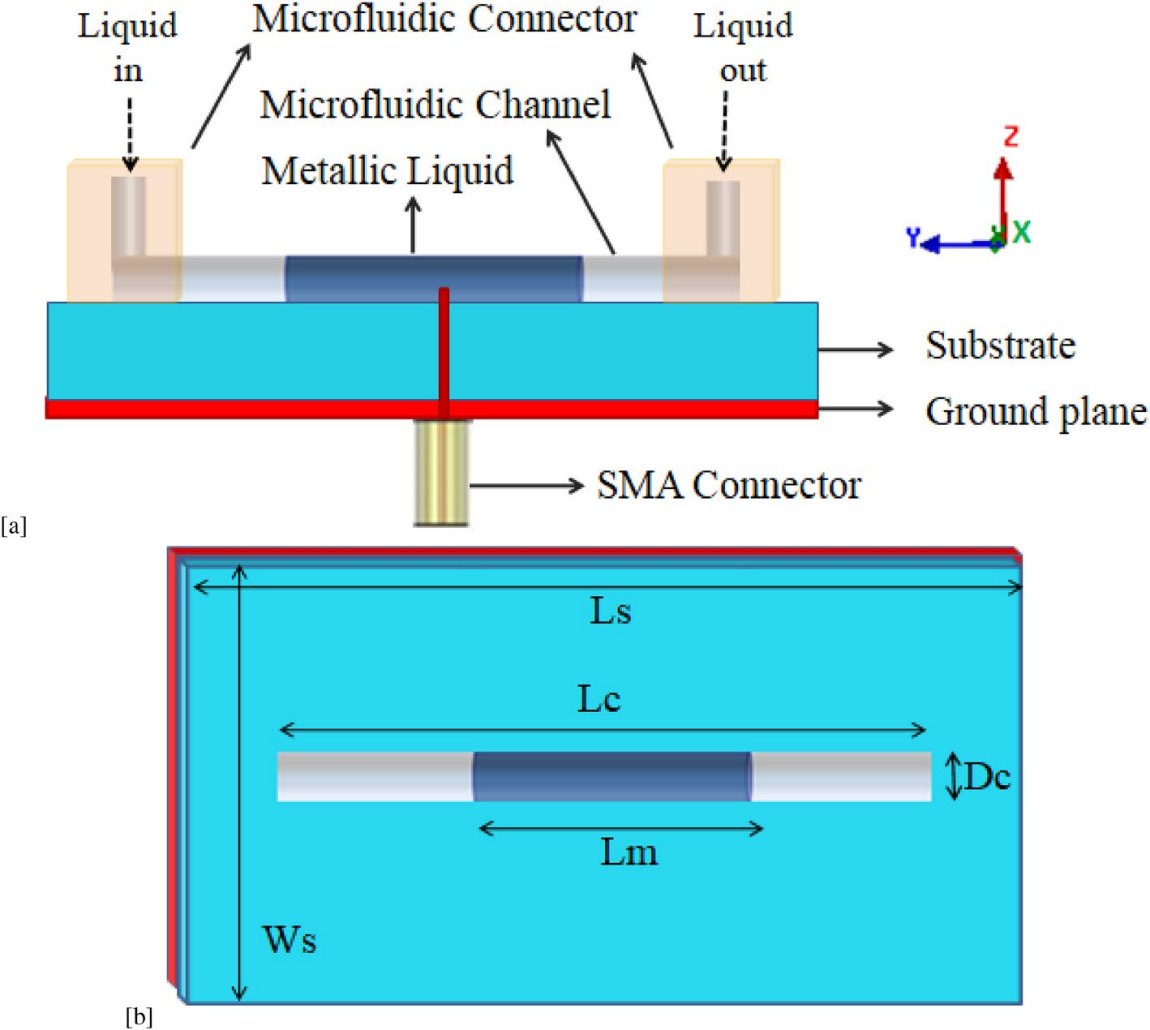
Schematic of the proposed metallic liquid antenna (**a**) Cross-sectional view and (**b**) 3D view.

Graphene was first isolated and characterized in 2004^21^. The superior properties of Graphene, including high electrical conductivity, high mechanical strength, adsorption capability, thermal resistance, etc., have led to a great deal of current research interest and a wide range of practical applications. Pristine Graphene has remarkable physical properties, including high electronic mobility ($\sim 250,000~ \hbox {cm}^{2}/\hbox {V}\,\hbox {s}$), excellent optical transparency (up to $\sim 97.7\%$), and high electrical and thermal conductivity (over 3000 W/m K). In addition, Graphene with no defects or impurities, has great strength, rigidity, and elasticity. It has a mechanical strength of $\sim 130~ \hbox {GPa}$ and an elasticity of $\sim 1.0 ~\hbox {Tpa}$. Graphene is $100\%$ pure and stable. Liquid Graphene is safe to use in industry and environmentally friendly. Currently, liquid-based Graphene is found in various applications^22^. The utilization of Graphene inks for flexible electronic applications has been the subject of extensive research in recent years^23^. For the first time, we employ a Graphene-conductive liquid for antenna designs in this work. Table 1 presents the material properties of metallic liquids for antennas.

## Results

We design, numerically analyze, and compare the electromagnetic performance of three different metallic liquid antennas made up of Mercury, EGaIn and Graphene metallic liquid in the Sub-6 GHz frequency in order to find a suitable metallic liquid for effective Sub-6 GHz antennas. A metallic liquid in a microfluidic channel is considered as the candidate antenna for comparison of the radiation performance for these three metallic liquids Mercury, EGaIn, and Graphene. These three antennas are realized by injecting a fixed volume ($\approx$ 1 ml) of metallic liquid into a PMMA microfluidic channel of length 35 mm over a metallic grounded LCP substrate of dimension $(50 \times 30 \times 3) ~\hbox {mm}^{3}$. For the proposed antenna structure, platinum metal is considered as a ground plane. Figure 1a, b illustrate the 3D and cross-sectional view of the proposed metallic liquid antennas. The dimensions of the antennas are optimized for 5.6 GHz operational frequency. Geometrical antenna dimensions for this frequency of antenna are presented in Table 2. Ansys HFSS (FEM-based Electromagnetic Solver) is used to simulate the antennas. These antennas are excited by using the center-fed single probe method. The ground plane (bottom layer) of the antennas is usually a sheet of metal that is electrically connected to the outside conductor of an SMA connector. From its bottom center, the feeding probe inserts into the metallic liquid and is connected electrically to the internal conductor of the SMA.

**Table 2:** Geometrical dimension of metallic liquid antenna.

Ls (mm)	Ws (mm)	Lc (mm)	Dc (mm)	Lm (mm)
50	30	35	3	12

**Figure 2:**
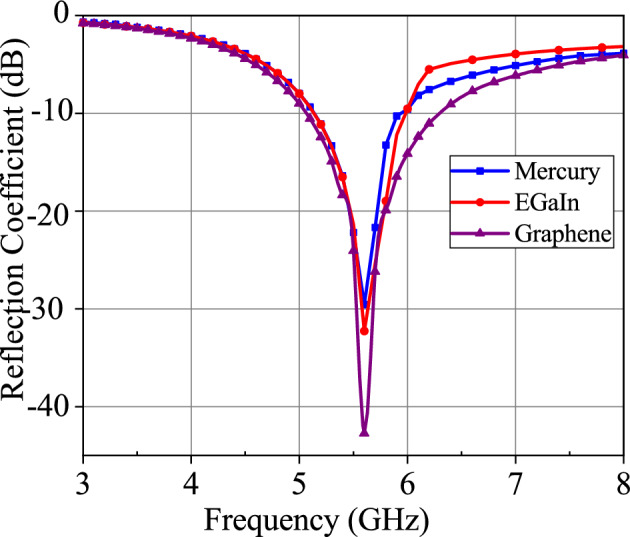
Reflection coefficient of Mercury, EGaIn and Graphene metallic liquid antennas.

**Figure 3:**
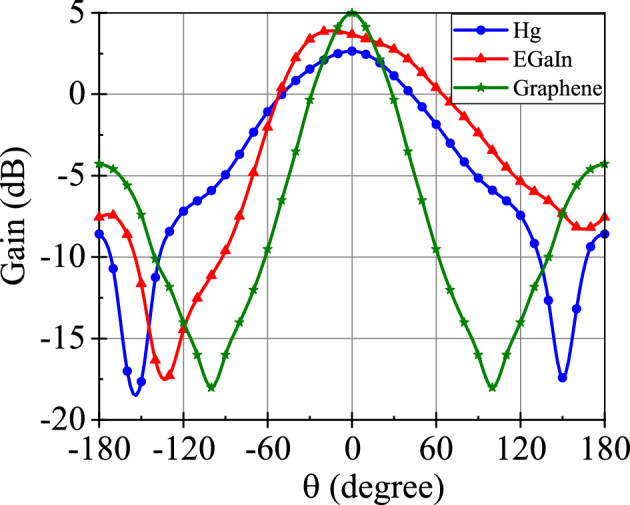
Radiation pattern of Mercury, EGaIn, and Graphene metallic liquid antennas at 5.6 GHz.

**Figure 4:**
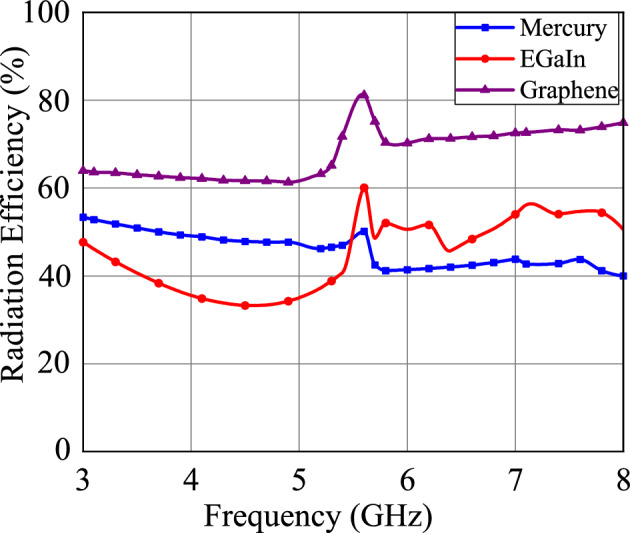
Radiation efficiency of Mercury, EGaIn, and Graphene metallic liquid antennas.

**Table 3:** Performance of Mercury, EGaIn, and Graphene Metallic Liquid antenna at 5.6 GHz.

Metallic liquid antenna	Reflection coefficient (dB)	Bandwidth (GHz)	Gain (dBi)	Radiation efficiency (%)
Mercury	− 29.5	0.75	2.6	50
EGaIn	− 32	0.58	3.6	60
Graphene	− 42	1.25	5.0	81

The reflection coefficients of Mercury, EGaIn, and Graphene metallic liquid antennas are shown in Fig. 2. It can be noticed that these three antennas resonate at the same frequency 5.6 GHz, whereas the reflection coefficients of these metallic liquid antennas are different, such as − 29.5 dB, − 32 dB, and − 42 dB for Mercury, EGaIn, and Graphene metallic liquid antennas, respectively. The Graphene metallic liquid antennas have a low reflection coefficient and wider 10-dB impedance bandwidth in comparison to conventional Mercury and EGaIn metallic liquid antennas.

The radiation patterns of these three antennas are shown in Fig. 3. Although the radiation patterns of the three antennas are almost similar, the difference in gain can be marked in Fig. 3. At 5.6 GHz frequency, Mercury, EGaIn, and Graphene metallic liquid antennas have gain of 2.6 dBi, 3.6 dBi, and 5.0 dBi, respectively. The radiation efficiency of antennas is illustrated in Fig. 4. The radiation efficiency of Graphene, EGaIn and Mercury metallic liquid antennas at 5.6 GHz are 81%, 60% and 50%, respectively. We can notice in Figs. 3 and 4 that the Graphene metallic liquid antenna has high gain and high radiation efficiency in comparison to Mercury and EGaIn metallic liquid antennas at operational frequency 5.6 GHz. The performance of these three metallic liquid antennas is summarized in Table 3. Owing to superior conductivity and fluidic properties, Graphene metallic liquid antenna performs better than Mercury and EGaIn liquid antennas. EGaIn liquid antennas perform better than Mercury liquid antennas.

Graphene is particularly best in terms of loss when used as an antenna and its performance is influenced by the loss characteristics of each material. The material’s conductivity influences the losses in the antenna. The less conductive the metal, the more loss takes place. From Table 1, it can be marked that Graphene has more conductivity than EGaIn and mercury. Therefore, Graphene metallic liquid antenna has a lower loss than EGaIn and mercury metallic liquid antenna.

The loss characteristics of each material in the operational frequency band further can be noticed in Fig. 2. This illustrates the loss of Mercury, EGaIn, and Graphene metallic liquid antennas. At an operational frequency 5.6 GHz, Graphene metallic liquid antennas have a low loss in comparison to Mercury and EGaIn metallic liquid antennas. The loss characteristic can also be marked from the radiation efficiency of the antenna in Fig. 4. Graphene metallic liquid antenna has high radiation efficiency in comparison to Mercury and EGaIn metallic liquid antennas at operational frequency 5.6 GHz. An antenna achieves high radiation efficiency if it has low loss. In the case of antenna with low radiation efficiency, more internal losses within the antenna takes place. Therefore, Graphene metallic liquid antenna has low loss compared to EGaIn and Mercury metallic liquid antenna.

## Discussion

The rapid growth of customer demands has necessitated the development of technologies and network capacity due to the major evolution of wireless technology from 1G to 6G. The sub-6GHz spectrum is used for optimal coverage in suburban and rural areas, whereas the mm-wave band is used in dense urban areas. The mm-wave networks are not feasible in rural and suburban areas due to their lack of range. In spite of the potential of multi-Gbps rates, several technical challenges exist in the mm-wave communication system^24^. The scope of mm-wave is likely to remain limited and will only be widely deployed in urban areas. With the growth of wireless generations and technologies, there has also been significant technological advancement in antenna design to meet the ever-growing requirements of customers^25^. The rapid growth of wireless communication systems necessitates the utilization of high-performance antennas in order to increase coverage and reduce the complexity of the system^26–29^. Henceforth, the 5G and 6G antennas should be efficient in terms of bandwidth, gain/directivity, efficiency, polarization, etc.

Unlike traditional conductors such as copper, metallic liquids have a flow property and lack of elasticity limit. This makes them ideal for applications that require mechanically flexible antennas. In general, conventional antennas are made with conductive metals like copper, which makes antennas very efficient but not flexible. By their nature, fluidic materials do not have any deformation limitations, making them the ideal candidates for flexible antenna applications. The liquid antenna has gained research interest due to its flexibility and reconfigurability feature. As far as metallic liquid antennas are concerned, the available metallic liquids are Mercury, EGaIn, and Graphene. For metallic liquid antenna design, Mercury is the first used metallic liquid and Graphene is the latest metallic liquid. The metallic liquid EGaIn, an alloy of gallium and tin metallic liquid also widely used for metallic liquid antenna design. The performance of metallic liquid antennas mainly depends on their fluidic and conductivity properties. In the sub-6 GHz spectrum, Graphene metallic liquid antenna with working frequency of 5.6 GHz performs better than EGaIn and Mercury metallic liquid antenna in terms of gain, bandwidth, reflection coefficient, and radiation efficiency.

Due to their inherent flexibility, liquid materials are a good substitute for stiff or solid conductors in flexible electronics. In addition to flexibility, another advantage of a metallic liquid antenna in a microfluidic channel is its reconfigurability. The fluid nature of liquid materials provides additional degrees of freedom to achieve better reconfigurability. Frequency reconfiguration and beam reconfiguration in metallic liquid antennas can be achieved by controlling the volume of liquid and the movement of liquid inside the microfluidic channel in different locations.

While offering unique advantages, metallic liquid antennas can indeed face challenges related to oxidation and residue formation when applied to microfluidic channels. This oxidation can create an oxide skin on the surface of the metallic liquid, which may affect the electrical properties of the antenna and potentially lead to the degradation of the antenna’s performance. Proper encapsulation or passivation techniques can be used to mitigate this issue. Treating the metallic liquid surface with a passivating material such as PMMA helps to prevent or reduce oxidation. Furthermore, metallic liquids can leave residues when they come into contact with certain materials. In microfluidic channels, these residues may lead to clogging or contamination of the channel, affecting the device’s performance. Properly selecting the materials used in the microfluidic channel helps mitigate residue formation. PMMA materials for the microfluidic channel are less susceptible to interaction with the metallic liquid and reduce the formation of residues and potential contamination.

The effects of oxidation and residues on liquid metal antennas in microfluidic channels vary depending on the specific liquid metal used, such as Mercury, EGaIn, and Graphene. Mercury is highly susceptible to oxidation. Oxidation can create an insulating layer on the surface of the metallic liquid, significantly reducing its electrical conductivity. This leads to a loss of antenna performance. Mercury leaves residues on the microfluidic channel walls over time. These residues can accumulate, leading to contamination and potential clogging of the channel. EGaIn can be generally preferred over mercury due to its reduced susceptibility to oxidation and the lower extent of residue formation. It has a less severe impact on electrical conductivity compared to mercury. EGaIn provides better antenna performance in terms of gain and radiation efficiency and reduces the risk of contamination and clogging. Graphene metallic liquids offer advantages in terms of oxidation mitigation and reduced residue formation. This helps in maintaining the electrical conductivity of the Graphene metallic liquid and antenna performance. Therefore, Graphene liquid metals provide better antenna performance in terms of reflection coefficient, gain and radiation efficiency compared to Mercury and EGaIn metallic liquid.

## Conclusion

This article presents a comparative analysis of Mercury, EGaIn, and Graphene metallic liquid material for sub-6 GHz antenna design in order to find a metallic liquid for designing efficient antennas in this frequency regime. The electromagnetic performance of a metallic liquid antenna made up of Mercury, EGaIn, and Graphene metallic liquid with 5.6 GHz resonant frequency is simulated and numerically analyzed in an EM simulator. The performance of these three antennas at the same working frequency is quite different because of their fluidic and conductivity properties. The antenna made up of Graphene liquid performs better than the antenna made up of Mercury and EGaIn metallic liquid in terms of gain, bandwidth, reflection coefficient, and radiation efficiency. The overall conclusion is the best suitability of Graphene metallic liquid for the design of metallic liquid antennas operating for sub-6 GHz systems.

## Method

The Ansys HFSS, a finite element method (FEM)-based electromagnetic (EM) solver, is used to validate the proposed designed antenna^30^. Three different metallic liquid antennas of Mercury, EGaIn, and Graphene with a resonant frequency of 5.6 GHz are simulated. Three metallic liquid antennas are realized by injecting a fixed volume ($\approx$ 1 ml) of metallic liquid into a PMMA microfluidic channel of length 35 mm over a metallic grounded LCP substrate of dimension $(50 \times 30 \times 3) ~\hbox {mm}^{3}$. The platinum metal is used as a ground plane for the proposed antenna structure. The volume of metallic liquid and dimensions of the antenna structures are optimized for 5.6 GHz operational frequency. In the EM simulation, Mercury ($\epsilon _{r}$ =1, tan $\delta$ = 0.001, $\sigma$ = $1.0 \times 10^{6}$ S/m) and EGaIn ($\epsilon _{r}$ = 3.3, tan $\delta$ = 0, $\sigma$ = $3.4 \times 10^{6}$ S/m) at operational frequency 5.6 GHz are used^30^. However, to model Graphene-based liquid, it is essential to model the conductive liquid with the surface conductivity $\sigma _s$ of Graphene in the operational frequency 5.6 GHz according to Kubo formalism^31^. Therefore, Graphene liquid in the EM simulator is modelled as conductive liquid with surface conductivity $\sigma _s$.

1 $$\begin{aligned} \begin{aligned} \sigma _s&= -j\frac{e^2K_BT}{\pi \hbar ^2 (\omega -j\tau ^{-1})} \left[ \frac{\mu _c}{K_BT}+2\ln \left( \exp {\left( -\frac{\mu _c}{K_BT} \right) }+1\right) \right] , \end{aligned} \end{aligned}$$

where *j* is the imaginary unit, $K_B$ is the Boltzmann’s constant, *e* is the electronic charge, *T* is the temperature, $\omega$ is the angular frequency, $\hbar$ is the reduced Planck’s constant, $\tau$ is the relaxation time and $\mu _c$ is the chemical potential.

The feeding mechanism of the proposed metallic liquid antenna can be illustrated with the help of Fig. 1. These antennas are excited by using the center-fed single probe method. Antennas with metallic liquid radiating elements that have narrow cross sections (such as a dipole) are excited at the middle of the antenna structure. The feed structure of a metallic liquid antenna in a microfluidic channel typically involves connecting the external conductor of the SMA adapter to the ground plane. The feeding probe is inserted into the metallic liquid from its bottom center and it is electrically connected to the inner conductor of the SMA, as depicted in Fig. 1. To ensure maximum radiation from the antenna, it is essential to achieve impedance matching. The antenna impedance matching behavior is shown in Fig. 2. It can be seen that three antennas antenna possess a well-matched resonant frequency at 5.6 GHz.

The fabrication feasibility of the proposed metallic liquid antenna can be explained with the help of Fig. 1a. Antennas with metallic liquid radiating elements that have narrow cross sections (such as a dipole) can be fabricated by simply injecting the metallic liquid into microfluidic channel. In this work, three antennas using Mercury, EGaIn, and Graphene metallic liquid can be realized by injecting metallic liquid into a PMMA microfluidic channel ($\epsilon _{r}$ = 2.55, tan $\delta$ = 0.002) over a metallic grounded LCP substrate ($\epsilon _{r}$ = 2.9, tan $\delta$ = 0.0025)^10,32^. The soft lithographic processes can be used to fabricate microfluidic channel^5,10,33^. The microfluidic channel of PMMA elastomer can be sealed with a thin and flat sheet of LCP-based substrate layer^10,34^. The position of metallic liquid in the microfluidic channel can be chosen as per the need of the desired result. The position of metallic liquid in different locations leads to modification in antenna results in terms of frequency and/or radiation pattern. In the present work, the position of metallic liquid is created at the center of the microfluidic channel.

The injection of metallic liquid at a certain position in the microfluidic channel can be possible in the following way. Initially, a syringe can inject the metallic liquid into the PMMA channel to fill the microfluidic that defines the radiating element. A bidirectional micropump unit is needed to reconfigure the physical length of the antenna by retracting a portion of the metallic liquid volume. Physical displacement of metallic liquid can be achieved through microfluidic techniques like pumping or electrowetting^34^. Digital microfluidics is also a new consideration for the physical displacement of metallic liquid in microfluidic channel^35^. Another way to set the metallic liquid at a certain position in the microfluidic channel is by filling the empty sections with de-ionized water^34^. Since the total volume of water is very small, the frequency/radiation pattern modifications and efficiency reduction due to the water and losses are not significant^34^.

In addition to flexibility, another advantage of a metallic liquid antenna in a microfluidic channel is its reconfigurability. The properties of the metallic liquid, such as its volume and shape, can be adjusted to tune the antenna’s operating frequency. The movement of liquid inside the microfluidic channel in different locations can reconfigure the radiation pattern. The antenna can achieve reconfigurability by changing the metallic liquid configuration within the microfluidic channel.

## Data Availability

All data generated or analyzed during this study are included in this manuscript.
